# Nipple adenoma in a female patient presenting with persistent erythema of the right nipple skin: case report, review of the literature, clinical implications, and relevancy to health care providers who evaluate and treat patients with dermatologic conditions of the breast skin

**DOI:** 10.1186/s12895-016-0041-6

**Published:** 2016-05-20

**Authors:** Gina P. Spohn, Shannon C. Trotter, Gary Tozbikian, Stephen P. Povoski

**Affiliations:** Division of Dermatology, Department of Internal Medicine, The Ohio State University Wexner Medical Center, Columbus, 43210 OH USA; Department of Pathology, The Ohio State University Wexner Medical Center, Columbus, 43210 OH USA; Division of Surgical Oncology, Department of Surgery, Arthur G. James Cancer Hospital and Richard J. Solove Research Institute and Comprehensive Cancer Center, The Ohio State University Wexner Medical Center, Columbus, 43210 OH USA

**Keywords:** Breast, Nipple, Adenoma, Papillomatosis, Adenomatosis, Proliferation, Erosive, Case report

## Abstract

**Background:**

Nipple adenoma is a very uncommon, benign proliferative process of lactiferous ducts of the nipple. Clinically, it often presents as a palpable nipple nodule, a visible nipple skin erosive lesion, and/or with discharge from the surface of the nipple skin, and is primarily seen in middle-aged women. Resultantly, nipple adenoma can clinically mimic the presentation of mammary Paget’s disease of the nipple. The purpose of our current case report is to present a comprehensive review of the available data on nipple adenoma, as well as provide useful information to health care providers (including dermatologists, breast health specialists, and other health care providers) who evaluate patients with dermatologic conditions of the breast skin for appropriately clinically recognizing, diagnosing, and treating patients with nipple adenoma.

**Case presentation:**

Fifty-three year old Caucasian female presented with a one year history of erythema and induration of the skin of the inferior aspect of the right nipple/areolar region. Skin punch biopsies showed subareolar duct papillomatosis. The patient elected to undergo complete surgical excision with right central breast resection. Final histopathologic evaluation confirmed nipple adenoma. The patient is doing well 31 months after her definitive surgical therapy.

**Conclusions:**

Since nipple adenoma represents a benign proliferative process of the nipple, complete surgical excision is curative. However, the coexistence of nipple adenoma and ipsilateral or contralateral breast cancer is well reported in the literature. The potential for a direct causal link or association of nipple adenoma and breast cancer cannot be fully excluded.

## Background

The accurate diagnosis of breast diseases is of paramount importance to both patients and clinicians. It is highly impactful on treatment planning, prognostication, and the resultant financial and psychosocial consequences. In the United States, breast cancer ranks second only to skin cancers among all new cancer cases diagnoses among women, with breast cancer representing 29 % of all new cancer case diagnoses among women [1]. In light of these staggering breast cancer statistics, it is important to recognize benign breast conditions (including conditions affecting the skin of the breast) which can clinically and histologically mimic malignant conditions of the breast. One such benign breast entity is nipple adenoma (NA).

NA is a very uncommon condition of the breast, primarily seen in middle-aged women, and representing a benign proliferative process of lactiferous ducts of the nipple [2–130]. Clinically, it often presents as a palpable nipple nodule, a visible nipple skin erosive lesion, and/or with discharge from the surface of the nipple skin. When NA is noted to have visibly eroded through the skin of the nipple, it can readily clinically mimic a case of mammary Paget’s disease of the nipple or an even more rare case of squamous cell carcinoma of the nipple. A nipple biopsy confirmation and subsequent complete surgical excision remain the gold standard for diagnosis and treatment of NA. However, more recently, alternate approaches have been suggested. New diagnostic tools include dermatoscopic examination (i.e., diascopy) [127], touch prep cytology [124], curettage/scrape cytology [117, 129] and fine needle aspiration [46, 47, 56, 70, 90, 124]. Alternate treatment interventions include Mohs micrographic surgery [76, 84, 98], nipple splitting enucleation of the NA [80, 116, 119], and cryotherapy [74].

It is likely that NA represents an under-recognized condition amongst any patient presenting with an abnormality of the skin of the nipple/areolar region. As such, patients may have symptoms for many months to many years before presenting to a health care provider for evaluation. Resultantly, the literature on NA has been somewhat limited, and has primarily consisted of multiple case reports and small case series, although a few larger case series do exist [3–130]. The purpose of our current case report is to present a comprehensive review of the available data on NA, as well as provide useful information to health care providers (including dermatologists, breast health specialists, and other health care providers) who evaluate patients with dermatologic conditions of the breast skin for appropriately clinically recognizing, diagnosing, and treating patients with NA.

## Case presentation

**Fig. 1:**
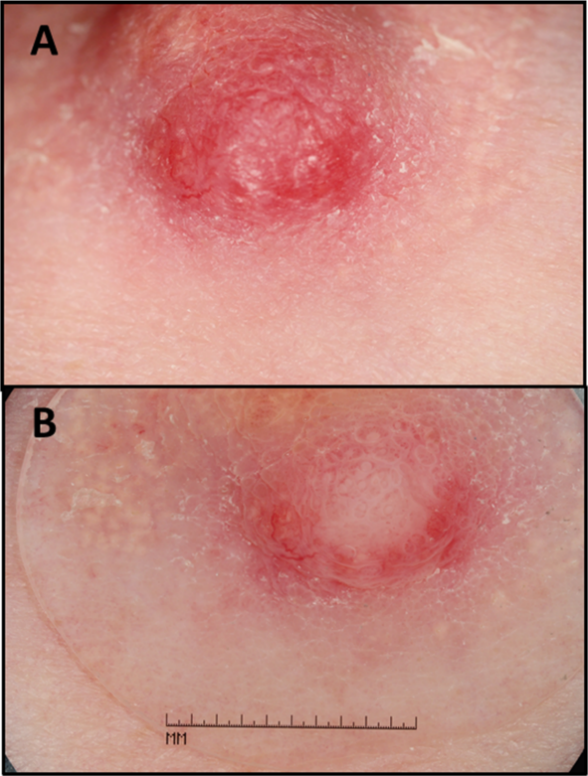
Initial clinical appearance of right nipple. **a** Close-up view of the initial appearance of the right nipple profile and surrounding areolar skin (only visualizing the lower one-half portion of the right nipple profile and surrounding areolar skin), with erythema and induration of the skin at the junction of the inferior aspect of the right nipple profile and surrounding areolar skin. **b** Diascopy examination was performed showing the dermatoscopic view of the same lesion of the right nipple profile, demonstrating red serpiginous and annular structures which are most prominent at the 6 o’clock position of the right nipple profile

A 53 year old Caucasian female with a past medical history of right eye choroidal melanoma presented with a one year history of erythema and induration of the skin at the junction of the inferior aspect of the right nipple profile and surrounding areolar skin (Fig. 1a). The patient had subsequently been treated with the application of topical steroids and topical antibiotics to the right nipple profile and surrounding areolar skin for the duration of approximately 5 months, and showed no clinical improvement. No palpable intraparenchymal breast masses were detected on clinical breast examination within either breast. A bilateral digital mammogram performed approximately seven months before presentation was within normal limits. Dermatoscopic findings revealed increased red serpiginous and annular structures most prominent at the 6 o’clock position of the right nipple profile (Fig. 1b).

An initial 3 mm punch biopsy of the skin at the junction of the inferior aspect of the right nipple profile and surrounding areolar skin was obtained by a dermatologist and histopathologic evaluation was reported to show subareolar sclerosing duct hyperplasia without abnormalities of the skin. Subsequently, one month later, a larger 6 mm punch biopsy was performed by a breast surgical oncologist to the same region of the right breast and histopathologic evaluation was reported to show adenosis and associated usual type ductal hyperplasia, consistent with subareolar duct papillomatosis. No atypia or malignancy was identified within either of the two sequential skin punch biopsy specimens. Repeat diagnostic digital mammography was performed on the patient during her evaluation by the breast surgical oncologist, and showed stable, benign-appearing right breast calcifications, and no suspicious mammographic findings within the right subareolar region or elsewhere within the right breast.

**Fig. 2:**
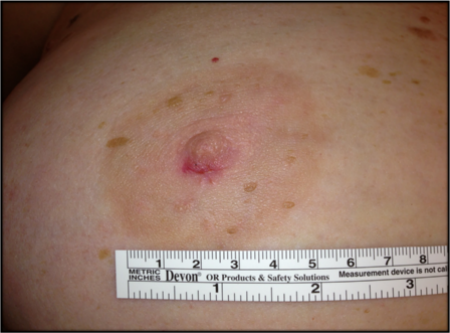
Subsequent appearance of the entire right nipple profile and surrounding areolar skin after the initial 3 mm skin punch biopsy and the subsequent 6 mm skin punch biopsy at the 6 o’clock position of the right nipple with surrounding erythema and induration

**Fig. 3:**
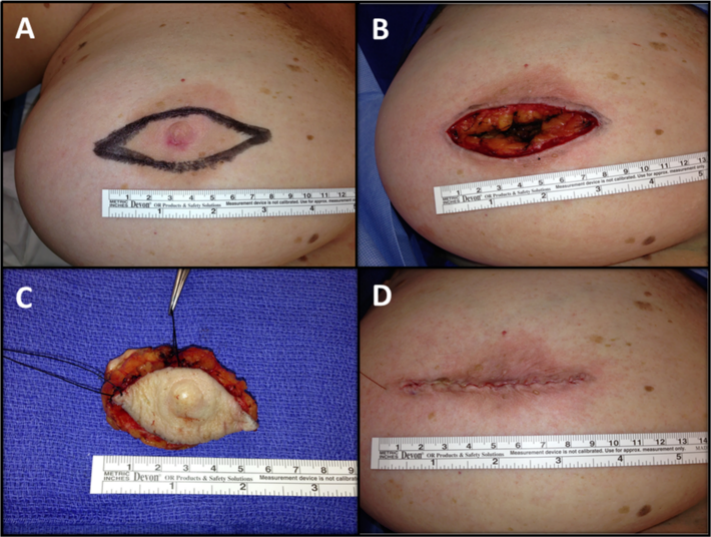
Surgical excision. **a** Planned elliptical-shaped surgical excision site for right central breast resection of right nipple profile and adjacent surrounding areolar skin prior to surgical excision. **b** Surgical excision bed after right central breast resection of right nipple profile and adjacent surrounding areolar skin. **c** Surgical excision specimen, consisting of right nipple profile, adjacent surrounding areolar skin, and superficial underlying breast and subcutaneous tissues. **d** Surgical closure of right central breast resection site without reconstruction of a “neo-nipple”, as per patient preference

The patient was subsequently taken to the operating room (Fig. 2), and elected to undergo a right central breast resection, consisting of surgical excision of the right nipple profile, adjacent surrounding areolar skin, and superficial underlying breast and subcutaneous tissues (Fig. 3a-c). The patient elected to simply have primary skin closure of her right breast surgical incision site, and without any attempt at cosmetic reconstruction of a right “neo-nipple” (Fig. 3d).

**Fig. 4:**
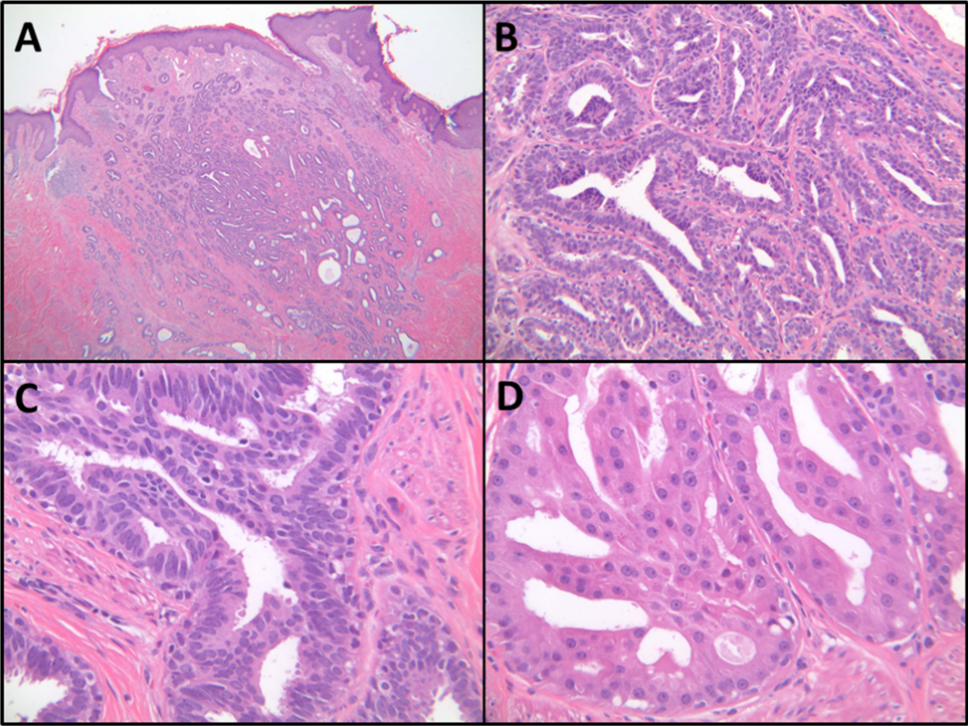
Histologic examination of the excised right nipple tissue. **a** Low Power (hematoxylin and eosin, 2×): Shows a circumscribed, compact aggregate of tubules within the nipple stroma and nipple skin dermis. **b** Medium Power (hematoxylin and eosin, 20×): Shows adenosis with proliferation of benign tubular and oval glands. **c** High Power (hematoxylin and eosin, 40×): Several glands show usual type ductal hyperplasia. **d** High Power (hematoxylin and eosin, 40×): Several glands show apocrine metaplasia

**Fig. 5:**
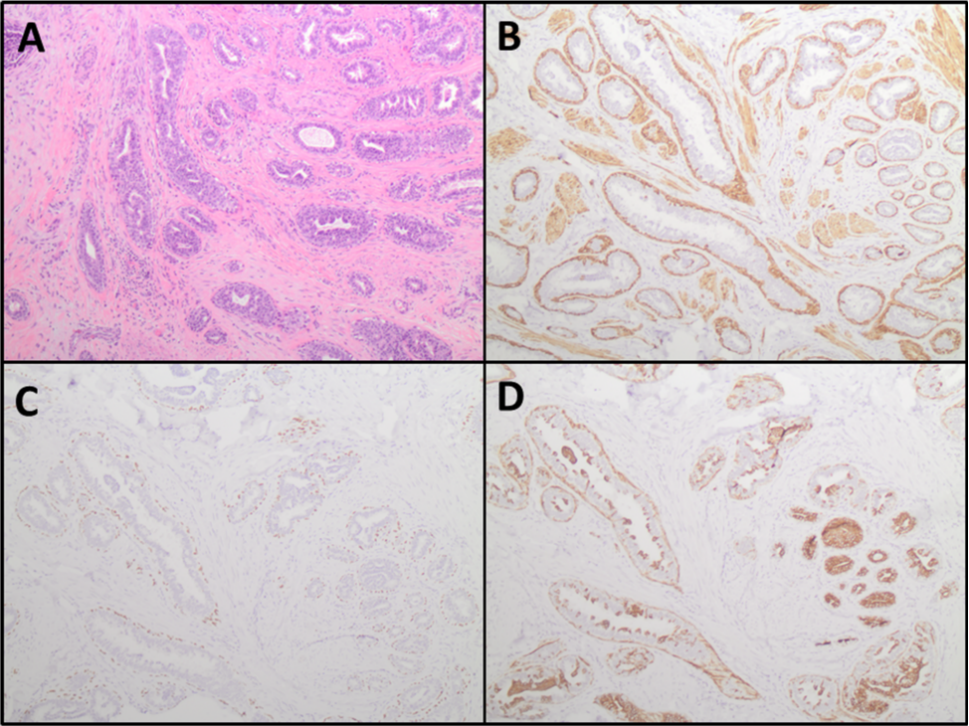
Immunohistochemical studies supporting the diagnosis of nipple adenoma. **a** Medium power (hematoxylin and eosin, 10×): Shows adenosis and usual type ductal hyperplasia. **b** Smooth muscle myosin heavy chain (10×) and (**c**) p63 (10×): Highlight the presence of myoepithelial cells surrounding benign glands. **d** CK5 (10×): Shows mosaic pattern of reactivity in foci of usual type ductal hyperplasia

Histopathologic evaluation by a breast-specific pathologist of hematoxylin and eosin stained sections from the right central breast resection specimen revealed a well-circumscribed, compact proliferation of tubular glands within the nipple stroma and nipple skin dermis (Fig. 4a). The lesion appeared centered in the reticular dermis, with focal extension into the papillary dermis. The overlying epidermis showed acanthosis, but was not directly involved by the lesion itself. Epidermal ulceration was not identified. At medium power, an adenosis pattern with proliferation of benign tubular glands was seen (Fig. 4b). At high power, several glands showed usual type ductal hyperplasia and apocrine metaplasia (Fig. 4c and d). A medium power hematoxylin and eosin stained section (Fig. 5a) and the corresponding immunohistochemical stained sections (Fig. 5b, c and d) are shown collectively in Fig. 5. Immunohistochemical stains for p63 (antibody BC4A4, BioCare Medical Inc., Concord, CA; Dilution 1:300 HIER, Bond Epitope Retrieval solution 1, Bond Autostainer) and smooth muscle myosin heavy chain (antibody SMMS-1, Dako, Carpinteria, CA; Dilution 1:350 HIER, Bond Epitope Retrieval solution 1, Bond Autostainer) confirmed the presence of myoepithelial cells surrounding the glands (Fig. 5b and c). CK5 (antibody XM26, Novocastra, Buffalo Grove, IL; Dilution 1:150 HIER, Bond Epitope Retrieval solution 2, Bond Autostainer) showed a mosaic pattern of reactivity in foci of usual type ductal hyperplasia (Fig. 5d). Therefore, a final pathologic diagnosis of NA was given. There was no histologic evidence of atypia or malignancy identified within the submitted specimen at the time of histopathologic evaluation.

The patient has continued regularly scheduled follow-up with her dermatologist and her surgical oncologist. At the current time, some 31 months after her definitive surgical therapy to her right breast, the patient remains without any evidence of any recurrent process within her right breast.

## Discussion/review

### Definition

NA is a benign proliferative process of lactiferous ducts of the nipple [2–130]. Historically, NA has been known by a variety of other names in the literature, including nipple duct adenoma, papillary adenoma of the nipple, florid papillomatosis of the nipple, florid adenomatosis of the nipple, erosive adenomatosis of the nipple, papillomatosis of the nipple, subareolar sclerosing duct hyperplasia of the nipple, subareolar duct papillomatosis of the nipple, and superficial papillary adenomatosis of the nipple. NA was first described by Jones [3] in 1955 as “florid papillomatosis of the nipple ducts”. Ten years later in 1965, Taylor and Robertson [15] argued that the name “adenoma of the nipple” be used to describe such tumors with adenomatous proliferation into nipple stroma with little to no proliferation into the lumen of the nipple ducts. They differentiated an adenoma of the nipple as a separate process from that of ductal papillomatosis of the nipple [15]. Later on in 1972, in a report of 65 patients, Perzin and Lattes [26] argued for the name “papillary adenoma of the nipple” to describe what had previously been reported as florid papillomatosis of the nipple, adenoma of the nipple, florid adenomatosis of the nipple, subareolar duct papillomatosis of the nipple, and erosive adenomatosis of the nipple. After decades of reporting on this entity of the nipple in a fashion that has both separated and lumped the various suggested terminologies, the current accepted definition decided upon by the 2012 WHO classification of breast tumors was designated as “nipple adenoma” (NA) [2].

### Histopathologic features

Histologically, NA can appear similar to other breast conditions (including syringomatous adenoma of the nipple, intraductal papilloma, adenomyoepithelioma, ductal carcinoma in situ, and invasive ductal carcinoma) as well as several dermatologic lesions (including syringoma of the skin, hidradenoma papilliferum, and syringocystadenoma papilliferum) [2, 15, 23, 26, 52, 118, 131]. The major histologic features of NAs are that they represent a ductal proliferation of glandlike structures within the stroma of the nipple, and generally have fairly well circumscribed borders but without encapsulation [8, 15, 23, 26, 52, 118]. Sclerosis/fibrosis may distort glands, mimicking an invasive growth pattern. Adenosis, cystic dilation, ductal hyperplasia, papillary hyperplasia, apocrine metaplasia, squamous metaplasia, and keratin cysts can be seen to varying degrees in NAs. Immunohistochemical stains can be useful to highlight the presence of two cell layers (i.e., epithelial and myoepithelial cells) [118, 132]. Specifically, p63, p40, calponin 1, h-caldesmon, CK5/6, CD10, or alpha smooth muscle actin and smooth muscle myosin can be used to highlight the presence of myoepithelial cells. Cytokeratin CK7 highlights the ductal epithelium and support the diagnosis. Recently, 5-hydroxymethylcytosine, an epigenetic modifier, has been suggested as a putative marker for NA [128].

The growth of NAs into the overlying dermis and epidermis, as well as erosion/ulceration through the epidermis is not infrequently seen [26, 52]. Interestingly, some cases which clinically appear to represent the erosion/ulceration of the NA through the epidermis are actually not due to erosion/ulceration of the NA through the epidermis, and instead represent the dilatation of major nipple ducts and the direct exposure of the papillomatous lesion lining those major nipple ducts to the surface of the nipple [26].

According to the *WHO Classification of Tumours of the Breast* [2], the 4 most common recognized histological subtypes of NA are: (1) adenosis type; (2) epithelial hyperplasia or papillomatosis type; (3) sclerosing papillomatosis or pseudo-infiltrating type; and (4) mixed type. The adenosis type shows proliferating glands extending from collecting ducts, is localized to the dermis, and typically lacks hyperkeratosis, inflammation, erosion, and/or ulceration. The papillomatosis type primarily has epithelial hyperplasia of the collecting duct epithelia and hyperplastic glandular ducts and is the type most commonly mistaken for mammary Paget’s disease of the nipple. In the sclerosing papillomatosis type, a pseudo-infiltrating pattern is distinguished by a prominence of proliferating epithelium into the stroma. Lastly, the mixed type may show features of any of the other three aforementioned subtypes. The patient presented in our current case report had both histologic evidence of adenosis and hyperplasia, most consistent with a mixed type of NA. It is our own personal opinion that most NAs will histologically display features in common across more than one of the aforementioned subtypes. Thus, the histological subtyping of NAs is somewhat arbitrary secondary to shared histologic features that can be seen within any given NA, and the resultant clinical relevance of the histological subtyping of NAs remains in question.

### Clinical presentation

NA most typically presents in women in their 4th and 5th decades of life [26, 52]. However, it has also been reported in men [2, 12, 14, 20, 31, 44, 90, 96, 104, 107, 112, 123], as well as throughout childhood [86, 99, 106]. While most cases of NA are unilateral, there have been rare reports of bilateral disease [22, 28]. The initial clinical presentation is most often that of nipple skin erosion, crusting, inflammation, erythema, itching, and/or associated pain of the nipple region [3–130]. The finding of serous and/or sanguineous discharge from the skin surface of the nipple profile is commonly reported as an initial presenting symptomatology and is generally secondary to the presence of a nipple skin erosive lesion. However, this serous and/or sanguineous discharge is often confused with genuine nipple discharge from the ducts within the nipple profile itself. In the more advanced presentations of NA, the nipple may become firm, nodular, and/or deformed. Clinically, NA may resemble mammary Paget’s disease of the nipple, squamous cell carcinoma of the nipple, eczema, psoriasis, or skin infection. Lastly, cases of NA have even rarely been reported to have arisen from a supernumerary mammary gland location [37, 109, 113].

### Diagnosis

The gold standard for making the most definitive final diagnosis of NA is histopathologic examination of a completely excised lesion [3–130]. However, nipple tissue biopsy with histopathological evaluation and confirmation prior to complete lesion excision is highly recommended. Imaging studies, including mammography and breast ultrasound are generally unable to provide adequate information for confirming the presence of NA due to the similarity in tissue density of the nipple to the surrounding skin and the underlying breast tissue [129]. However, digital mammography should always be considered for ruling out any mammographic abnormalities in the underlying breast tissue when a patient presents with any significant nipple symptomatology. Breast ultrasound has been reported by some to be a potential useful tool for identifying NA, as based upon the findings of homogenous echogenicity and hypervascularity [79, 103, 105, 116], while others have found its use limited and inconclusive [129]. Breast magnetic resonance imaging has also been reported to allow for characterization of NA [81, 95, 111]. In addition to microscopic tissue section examination of excised tissues using hematoxylin and eosin and immunohistochemical techniques, cytologic examination using touch prep cytology [124], curettage/scrape cytology [117, 129] and fine needle aspiration [46, 47, 56, 70, 90, 124] has also been evaluated. Ozaki et al. [124] reported four cases of NA in which cytologic examination by brush cytology, aspiration cytology, and/or tumor imprint cytology were used to aide in the benign or malignant characterization of such lesions. All four of these NA cases showed a small to large papillary cluster of epithelial cells, round to oval nuclei, and with three of the four cases also having attached myoepithelial cells.

Dermatoscopic examination (i.e., diascopy) has also been proposed as a potentially useful diagnostic modality [127]. In 2015, Takashima et al. [127], reported a single case of NA in a 57-year old Japanese woman presenting with erosive erythema. Dermatoscopic evaluation of the nipple lesion showed linear, cherry-red structures thought to be representative of neoplastic tubular luminal openings of the NA. Interestingly, the dermatoscopic photography of the patient presented in our current case report demonstrated red serpiginous and annular structures rather than linear cherry-red structures as reported by Takashima et al. [127].

### Treatment

It is universally agreed upon that complete surgical excision of the entire NA is important for preventing local recurrence [3–130]. As in our particular case, complete surgical excision has traditionally been accomplished by resection surgical excision of the right nipple profile, adjacent surrounding areolar skin, and superficial underlying breast and subcutaneous tissues [38, 118, 129]. However, more limited forms of complete surgical excision of the entire NA have been reported using a wedge resection technique [93, 94, 129], as well as a nipple splitting enucleation technique via a trans-nipple longitudinal incision made down through the long axis of the nipple profile to expose and extract the NA [80, 116, 119]. Likewise, Mohs micrographic surgery has been reported to be successfully used for NA excision and is thought to be curative [76, 84, 98]. Lastly, cryotherapy has been reported as a novel technique for eradication of a NA [74].

The potential for local recurrence of NA is always a concern with utilization of any of these more limited forms of complete lesion removal. When a NA grows from the nipple stroma and into the overlying dermis and epidermis or erodes through the epidermis, more limited forms of surgical excision, such as the nipple splitting enucleation technique, should not be considered. In such cases, complete excision of all involved nipple skin should be undertaken to assure complete lesion removal and to minimize the risk of local recurrence of the NA with the remaining nipple profile. In the case presented herein, in which the nipple adenoma clinically appeared to involve the nipple skin and histological was shown to involve the nipple dermis, our patient elected for complete surgical excision, primary skin closure, and no attempt at cosmetic reconstruction of a right “neo-nipple”. Since NA represents a benign proliferative process of the nipple, complete surgical excision is curative. It should be emphasized that any patient with a history of NA should be encouraged to maintain regular breast follow-up with continuation of annual clinical breast exams by their healthcare providers and annual digital screening mammography after successful NA removal.

### Is there an association between NA and breast cancer?

It is well documented that incidental breast cancer has been detected at the time of the excision of a NA [2, 8, 12, 24, 26, 27, 48, 49, 52, 53, 57, 67, 75, 87, 121, 130]. While most of these incidental breast cancers are found at the time of the initial NA excision, there are rare cases in which breast cancer has been reported at the site where a NA was previously biopsied or excised [2, 24, 36, 53, 121]. Eusebi and Lester reported that 24 of 173 (13.9 %) cases of NA were associated with breast cancer [2]. Likewise, Rosen and Caicco reported that 9 of 51 (17.6 %) cases of NA were associated with breast cancer [52]. Nevertheless, it remains unclear as to whether the presence of a NA represents a risk factor the subsequent development of breast cancer. Additionally, it is unknown whether the incidence of NA is greater in patients with a positive family history of breast cancer [26, 52]. The coexistence of NA and ipsilateral or contralateral breast cancer has been reported in surgical specimens of breast tissue excised at the time of breast cancer surgery [8, 12, 24, 26, 27, 48, 49, 52, 57, 67, 75, 87, 121, 130]. When ipsilateral NA and carcinoma are synchronously observed within a breast, they most often represent two independent lesions, with the site of carcinoma being located at a distinct and separated geographic location from the site of the NA. Furthermore, the relative incidence of NA in patients with breast cancer versus patients without breast cancer is not known. Despite these previously suggested associations, NA is itself not a malignant lesion of the breast. Nevertheless, a definitive association of NA with the subsequent development of breast cancer, as well as a direct causal link for the transformation of a NA into a later developing breast cancer process, cannot be fully excluded.

## Conclusions

In summary, NA is a benign proliferative lesion of the nipple. NA can be an important clinical mimic of mammary Paget’s disease of the nipple. If sclerotic, NA may even mimic invasive carcinoma histologically. The coexistence of NA and ipsilateral or contralateral breast cancer is well reported in the literature. In this regard, the potential for a direct causal link or association of NA and breast cancer cannot be fully excluded. Nipple tissue biopsy with histopathological evaluation is the current gold standard for diagnosis, but tools such as dermatoscopy and cytology have been proposed as less invasive diagnostic modalities. The standard-of-care treatment for NA is complete surgical excision, but alternate treatment interventions, such Mohs micrographic surgery, nipple splitting enucleation, and cryotherapy, have been used successfully in reported cases. Since NA is an uncommon and likely under-recognized phenomenon, it is important to continue reporting on new NA cases and to closely follow those patients over time. Such an approach may be useful for allowing us to continue to learn more about its natural history and for attempting to clarify the question of any potential direct causal link or association of NA and breast cancer. In light of our inability to exclude a direct causal link or association of NA and breast cancer, it is very reasonable to encourage patients with a history of NA to maintain regular breast follow-up with continuation of annual clinical breast exams by their healthcare providers and annual digital screening mammography after successful NA removal.

## Declarations

### Consent

Written informed consent was obtained from the patient for publication of this case report and any accompanying images. A copy of the written consent is available for review by the editor of this journal.

## Abbreviations

NA: nipple adenoma
